# Acute kidney injury among critically ill patients with pandemic H1N1 influenza A in Canada: cohort study

**DOI:** 10.1186/1471-2369-14-123

**Published:** 2013-06-13

**Authors:** Sean M Bagshaw, Manish M Sood, Jennifer Long, Robert A Fowler, Neill KJ Adhikari

**Affiliations:** 1 Division of Critical Care Medicine, Faculty of Medicine and Dentistry, University of Alberta, 3C1.12 Walter C Mackenzie Centre, 8440-112 St NW, Edmonton, AB T6G 2B7, Canada; 2 Division of Nephrology, University of Manitoba, 409 Tache Avenue, Winnipeg, MB R2N 2A6, Canada; 3 Department of Critical Care Medicine, Sunnybrook Health Sciences Centre, 2075 Bayview Avenue, Room D1.08, Toronto, ON M4N 3M5, Canada; 4 Department of Critical Care Medicine and Sunnybrook Research Institute, Sunnybrook Health Sciences Centre and University of Toronto, 2075 Bayview Avenue, Room D1.08, Toronto, ON M4N 3M5, Canada; 5 Department of Medicine, Sunnybrook Health Sciences Centre and University of Toronto, 2075 Bayview Avenue, Room D4.78, Toronto, ON M4N 3M5, Canada

**Keywords:** Acute kidney injury, Renal replacement therapy, Influenza, Critical illness, Mortality, Resource utilization

## Abstract

**Background:**

Canada’s pandemic H1N1 influenza A (pH1N1) outbreak led to a high burden of critical illness. Our objective was to describe the incidence of AKI (acute kidney injury) in these patients and risk factors for AKI, renal replacement therapy (RRT), and mortality.

**Methods:**

From a prospective cohort of critically ill adults with confirmed or probable pH1N1 (16 April 2009–12 April 2010), we abstracted data on demographics, co-morbidities, acute physiology, AKI (defined by RIFLE criteria for Injury or Failure), treatments in the intensive care unit, and clinical outcomes. Univariable and multivariable logistic regression analyses were used to evaluate the associations between clinical characteristics and the outcomes of AKI, RRT, and hospital mortality.

**Results:**

We included 562 patients with pH1N1-related critical illness (479 [85.2%] confirmed, 83 [14.8%] probable]: mean age 48.0 years, 53.4% female, and 13.3% aboriginal. Common co-morbidities included obesity, diabetes, and chronic obstructive pulmonary disease. AKI occurred in 60.9%, with RIFLE categories of Injury (23.0%) and Failure (37.9%). Independent predictors of AKI included obesity (OR 2.94; 95%CI, 1.75-4.91), chronic kidney disease (OR 4.50; 95%CI, 1.46-13.82), APACHE II score (OR per 1-unit increase 1.06; 95%CI, 1.03-1.09), and P_a_O_2_/F_i_O_2_ ratio (OR per 10-unit increase 0.98; 95%CI, 0.95-1.00). Of patients with AKI, 24.9% (85/342) received RRT and 25.8% (85/329) died. Independent predictors of RRT were obesity (OR 2.25; 95% CI, 1.14-4.44), day 1 mechanical ventilation (OR 4.09; 95% CI, 1.21-13.84), APACHE II score (OR per 1-unit increase 1.07; 95% CI, 1.03-1.12), and day 1 creatinine (OR per 10 μmol/L increase, 1.06; 95%CI, 1.03-1.10). Development of AKI was not independently associated with hospital mortality.

**Conclusion:**

The incidence of AKI and RRT utilization were high among Canadian patients with critical illness due to pH1N1.

## Background

In the spring of 2009, the world experienced a pandemic due to novel swine-origin influenza A (pH1N1) virus, with more than 17,000 deaths [1]–[6]. In Canada, there were two major waves of pH1N1 in the spring and fall of 2009 that led to 8,678 hospitalizations, of which 1,473 (17.0%) required intensive care unit (ICU) admission [7]. By April 2010, there were approximately 428 deaths (4.9%; 12.7 per million population) attributable to pH1N1 infection, the 12th highest pH1N1-attributable mortality worldwide [7].

Of particular concern among pH1N1 patients was the high risk of ICU admission for respiratory failure [7,8]. Several studies have described the impact of pH1N1 on ICU resources, with an emphasis on respiratory failure, mechanical ventilation, and extracorporeal life support (ECLS) [9,10]. Fewer studies have focused on non-pulmonary organ dysfunction, specifically acute kidney injury (AKI) and its resource implications. AKI commonly accompanies critical illness and independently increases risks of death, prolonged length of stay, and development of new chronic kidney disease (CKD) among survivors [11,12]. Recently, reports have described the incidence and outcomes of AKI among critically ill adult and pediatric pH1N1 patients [13]–[28]. Many studies, however, were limited by design (case reports [14,17,20,22,24,28] or small case series [13,15,18,21,27]), small sample size [16,19,25], or single-center enrollment [13,15,16,18,21]. There have been few larger scale prospective multi-center cohorts [19,23,26], none of which described the Canadian pandemic.

Accordingly, we examined a prospective multi-centre Canadian cohort of critically ill patients and confirmed or probable pH1N1 infection [2] to primarily describe the incidence and severity of AKI and secondarily determine the rates of RRT utilization along with risk factors for AKI, RRT, and mortality.

## Methods

This study was approved by the Research Ethics Board at Sunnybrook Health Sciences Centre, Toronto on 30 April 2009 (file #130-2009), and at all participating sites. All Research Ethics Boards waived the requirement for individual informed consent. The reporting of this study follows recent guidelines [29].

### Study design, setting, and participants

This cohort study is a secondary analysis of prospectively collected data from a multi-centre inception cohort of all adults (age >18 years), admitted to any of 51 Canadian ICUs, with confirmed or probable influenza A (pH1N1)-related critical illness, from 16 April 2009 to 12 April 2010 [2]. Members of the Canadian Critical Care H1N1 Collaborative are listed in Additional file 1. The 51 ICUs that agreed to collect data for this study constituted a subset of the 286 Canadian ICUs that provide mechanical ventilation (R. Fowler, personal communication). Data on a subset of 50 patients in this cohort, all from Manitoba, Canada, were published previously [27].

### Data collection

Data from patients’ medical records were captured on standardized case-report forms, as described elsewhere [2]. Briefly, we collected data on dates and times of hospital and ICU admission and discharge, demographics, co-morbid diseases, acute physiology, laboratory parameters, illness severity (APACHE II [Acute Physiology and Chronic Health Evaluation II] [30] and SOFA [Sequential Organ Failure Assessment] [31] scores), treatment intensity (including mechanical ventilation, inotrope and vasopressor support, and RRT, with each determined on days 1, 3, 7, 14, and 28 of ICU admission), and vital status at hospital discharge. The comorbid condition of obesity was defined as body mass index ≥30 kg/m^2^. Diagnostic tests, including scheduled serum creatinine levels, were not mandated by the study protocol.

### Cohort definitions

Patients with pH1N1 were included if confirmed or probable according to case definitions of the World Health Organization and the Canadian National Microbiology Laboratory [32]. Critical illness was defined by: 1) admission to an ICU and either 2) receipt of mechanical ventilation (invasive or non-invasive) or 3) receipt of inotrope or vasopressor infusion [2]. Chronic kidney disease (CKD) was defined as pre-morbid baseline serum creatinine ≥1.5 times the upper limit of normal. End-stage kidney disease was defined as outpatient dialysis dependence preceding ICU admission.

### Outcome definitions

The primary outcome was AKI, defined according to the RIFLE (Risk; Injury; Failure; Loss; End-stage kidney disease) classification scheme [33]. Patients were classified according to the worst RIFLE category achieved while in ICU, with RIFLE categories Injury and Failure used to define AKI. Categories were determined on days 1, 3, 7, 14, and 28 of ICU admission, using both creatinine and 24-hour urine output on those days, with baseline glomerular filtration rate estimated as 75 ml/min/1.73 m^2^[33]. Because this assumption may overestimate the incidence of AKI, we determined RIFLE categories using two additional approaches in sensitivity analyses: (1) assuming ICU day 1 creatinine as the baseline, and (2) assuming the lowest creatinine in the ICU as the baseline, except for patients who received RRT, who were all assumed to be in the Failure category. Both approaches would tend to underestimate the incidence of AKI because any creatinine measured in the ICU in a patient not on RRT is unlikely to be lower than baseline.

Secondary outcomes included utilization of RRT (any of continuous renal replacement therapy, slow low-efficiency hemodialysis, intermittent hemodialysis); hospital mortality; and lengths of ICU and hospital stay.

### Statistical analysis

The study sample size was based on the number of patients with pH1N1-related critical illness admitted to a study ICU during the period of pH1N1 activity. Continuous variables were summarized as means (standard deviations) or medians (quartile 1 [Q1] - quartile 3 [Q3]) as appropriate and categorical variables as proportions. We used Student’s two-sample t-test or Wilcoxon rank sum test to compare continuous variables and chi-square or Fisher’s exact test for categorical variables. Univariable and multivariable logistic regression were used to evaluate associations between clinical characteristics and the outcomes of AKI, RRT, and hospital mortality. Age and sex were included in all models, AKI was forced into the mortality model, and other explanatory variables were included if p ≤0.2 by univariable analysis. We used univariable analyses to screen potential variables because we anticipated having too few events to include all variables in the initial model. All selected variables initially entered in the multivariable models were retained. We excluded variables if data were missing for more than 15% of the study sample. Interaction terms were not considered. There was no evidence of multi-collinearity in any of the multivariable models (all correlations <0.8). We examined model calibration using the Hosmer-Lemeshow test; no model demonstrated lack of goodness-of-fit. We considered p < 0.05 to be statistically significant. All analyses were performed using SAS version 9.2 (SAS Institute Inc, Cary, USA).

## Results

Overall, 756 consecutive critically ill patients with confirmed or probable pH1N1 entered the cohort. For this study, 194 (25.7%) participants were excluded due to missing eligibility criteria (n = 2) or age (n = 1), pediatric status (n = 62), pre-admission dialysis dependence (n = 20), or insufficient data to assign a RIFLE category (n = 109). The remaining 562 (74.3%) patients (479 [85.2%] with confirmed and 83 [14.8%] with probable pH1N1) were included. To investigate selection bias, we compared the study sample and patients excluded due to insufficient data to assign RIFLE category (n = 109). Excluded patients had a shorter duration of symptoms before hospital and ICU admission, were less likely to be obese, and had lower treatment intensity, with fewer receiving mechanical ventilation, antiviral therapy, and RRT; they also had shorter lengths of ICU and hospital stay (Table 1).

**Table 1:** Comparison of participants included versus excluded due to missing data

**Characteristic**	**Overall**	**Included**	**Excluded**	**P value**
	**(n** = **671)**	**(n** = **562)**	**(n** = **109)**	
Age (yr)	47.9 (15.2)	48.0 (15.0)	47.1 (16.4)	0.55
Female sex	363 (54.1)	300 (53.4)	63 (57.8)	0.40
Ethnicity (n=652, 555, 97)				
White/Caucasian	378 (58.0)	327 (58.9)	51 (52.6)	0.41
Unknown/Mixed/Other	143 (21.9)	117 (21.1)	26 (26.8)	
Aboriginal	85 (13.0)	74 (13.3)	11 (11.3)	
Asian	46 (7.1)	37 (6.7)	9 (9.3)	
Any co-morbidity	607 (90.5)	513 (91.3)	94 (86.2)	0.10
Obesity	167 (24.9)	150 (26.7)	17 (15.6)	0.01
Diabetes mellitus	170 (25.3)	146 (26.0)	24 (22.0)	0.38
Chronic obstructive pulmonary disease	119 (17.7)	98 (17.4)	21 (19.3)	0.65
Alcohol abuse	83 (12.4)	74 (13.2)	9 (8.3)	0.15
Chronic kidney disease	56 (8.4)	48 (8.5)	8 (7.3)	0.68
H1N1 status				
Confirmed	574 (85.5)	479 (85.2)	95 (87.2)	0.60
Probable	97 (14.5)	83 (14.8)	14 (12.8)	
Days of symptoms pre-hospital (n = 635, 532, 103)	4.0 (2.0-7.0)	4.0 (2.0-7.0)	3.0 (1.0-6.0)	0.01
Days of symptoms pre-ICU (n = 653, 549, 104)	5.0 (3.0-8.0)	5.0 (3.0-8.0)	4.5 (2.5-7.0)	0.04
**Day 1 support, physiology, and laboratory values**				
APACHE II score (n = 594, 508, 86)	20.8 (9.8)	21.1 (9.2)	19.1 (13.1)	0.19
SOFA score (n = 411, 361, 50)	11.2 (3.7)	11.2 (3.8)	10.9 (3.6)	0.61
Invasive mechanical ventilation (n = 652, 553, 99)	431 (66.1)	384 (69.4)	47 (47.5)	<0.0001
Inotrope or vasopressor	281 (41.9)	251 (44.7)	30 (27.5)	0.0009
Mean arterial pressure (mmHg) (n = 613, 522, 91)	74.1 (18.7)	74.4 (18.9)	72.5 (17.4)	0.37
Heart rate (beats/min) (n = 504, 433, 71)	104.1 (24.0)	104.5 (24.1)	101.5 (23.3)	0.33
Temperature (°C) (n = 472, 405, 67)	37.6 (1.3)	37.6 (1.2)	37.5 (1.8)	0.48
P_a_O_2_/F_i_O_2_ (n = 570, 496, 74)	160 (120)	153 (96)	212 (215)	0.02
CK (U/L) (n = 270, 230, 40)	272 (91-776)	293 (91-835)	224 (87-663)	0.41
CK > 5000 IU/L (n = 270, 230, 40)	15 (5.6)	13 (5.7)	2 (5.0)	1.00
WBC (10^9^/L) (n = 471, 409, 62)	9.7 (6.5)	9.5 (6.5)	10.6 (7.0)	0.24
Albumin (g/L) (n = 315, 275, 40)	26.6 (7.2)	26.2 (7.0)	28.7 (8.0)	0.04
Platelets (10^9^/L) (n = 608, 520, 88)	190 (96)	188 (96)	204 (95)	0.16
Urea (mmol/L) (n = 89, 76, 13)	10.2 (19.7)	10.5 (21.0)	8.4 (9.4)	0.56
Creatinine (μmol/L) (n = 626, 537, 89)	125 (120)	124 (120)	131 (119)	0.59
Urine output (mL/24 hr) (n = 489, 431, 58)	1440 (1082)	1439 (1055)	1446 (1278)	0.97
**Interventions (ICU day 1, 3, 7, 14, or 28)**				
Renal replacement therapy (n = 670, 561, 109)	91 (13.6)	85 (15.2)	6 (5.5)	0.007
Invasive mechanical ventilation (n = 669, 562, 107)	568 (84.9)	499 (88.8)	69 (64.5)	<0.0001
Inotropes or vasopressors	373 (55.6)	339 (60.3)	34 (31.2)	<0.0001
Antiviral treatment	620 (92.4)	530 (94.3)	90 (82.6)	<0.0001
**Outcome**				
Days of ICU stay (n = 640, 539, 101)	11.0 (6.0-19.0)	11.0 (7.0-20.0)	5.0 (2.0-13.0)	<0.0001
Days of hospital stay (n = 626, 523, 103)	18.0 (11.0-34.0)	19.0 (12.0-35.0)	12.0 (5.0-24.0)	<0.0001
Hospital mortality (n = 649, 545, 104)	142 (21.9)	113 (20.7)	29 (27.9)	0.11

Patients described in this table were excluded because of insufficient data to determine the RIFLE classification. For each variable, we specify (n) with data if it differs from the total in the first row. Categorical data are presented as n (%) and continuous data as mean (SD) or median (Q1-Q3).

Patients had a mean (SD) age of 48.0 (15.0) years, 53.4% were female, 13.3% were aboriginal, and 91.3% had at least one co-morbid disease, with obesity (26.7%), diabetes mellitus (26.0%), and chronic obstructive pulmonary disease (17.4%) being most common (Table 2). Patients had high illness severity and burden of organ dysfunction (APACHE II score 21.1 [9.2]; day 1 SOFA score 11.2 [3.8]). On the first ICU day, 69.4% received mechanical ventilation and 44.7% received an inotrope or vasopressor (Table 3).

**Table 2:** **Baseline characteristics of critically ill pH1N1 patients**, **stratified by AKI**

**Characteristic**	**Overall**	**AKI**	**No AKI**	**P value**
	**(n** = **562)**	**(n** = **342)**	**(n** = **220)**	
Age (yr)	48.0 (15.0)	49.1 (15.1)	46.4 (14.8)	0.03
Female sex	300 (53.4)	177 (51.8)	123 (55.9)	0.34
Ethnicity (n=555, 336, 219)				0.23
White/Caucasian	327 (58.9)	204 (60.7)	123 (56.2)	
Unknown/Mixed/Other	117 (21.1)	68 (20.2)	49 (22.4)	
Aboriginal	74 (13.3)	47 (14.0)	27 (12.3)	
Asian	37 (6.7)	17 (5.1)	20 (9.1)	
Any co-morbidity	513 (91.3)	316 (92.4)	197 (89.6)	0.24
Obesity	150 (26.7)	118 (34.5)	32 (14.6)	<0.0001
Diabetes mellitus	146 (26.0)	107 (31.3)	39 (17.7)	0.0003
Chronic obstructive pulmonary disease	98 (17.4)	56 (16.4)	42 (19.1)	0.41
Alcohol abuse	74 (13.2)	41 (12.0)	33 (15.0)	0.30
Chronic kidney disease	48 (8.5)	44 (12.9)	4 (1.8)	<0.0001
H1N1 status				
Confirmed	479 (85.2)	291 (85.1)	188 (85.5)	0.90
Probable	83 (14.8)	51 (14.9)	32 (14.6)	
Days of symptoms pre-hospital (n = 532, 326, 206)	4.0 (2.0-7.0)	4.0 (2.0-7.0)	4.0 (2.0-7.0)	0.67
Days of symptoms pre-ICU (n = 549, 336, 213)	5.0 (3.0-8.0)	5.0 (3.0-8.0)	5.0 (3.0-8.0)	0.66

AKI is defined by the RIFLE Injury or Failure categories and no AKI by the RIFLE None or Risk categories.

Categorical data are presented as n (%) and continuous data as mean (SD) or median (Q1-Q3). For each variable, we specify (n) with data if it differs from the total in the first row.

**Table 3:** **Clinical course and outcomes of critically ill pH1N1 patients**, **stratified by AKI**

	**Overall**	**AKI**	**No AKI**	**P value**
	**(n** = **562)**	**(n** = **342)**	**(n** = **220)**	
**Day 1 support, physiology, and laboratory values**				
APACHE II score (n = 508, 306, 202)	21.1 (9.2)	23.0 (9.6)	18.1 (7.6)	<0.0001
SOFA score (n = 361, 216, 145)	11.2 (3.8)	12.0 (3.8)	9.7 (3.2)	<0.0001
Invasive mechanical ventilation (n = 553, 338, 215)	384 (69.4)	241 (71.3)	143 (66.5)	0.23
Inotrope or vasopressor	251 (44.7)	164 (48.0)	87 (39.6)	0.05
Mean arterial pressure (mmHg) (n = 522, 315, 207)	74 (19)	73 (19)	76 (19)	0.14
Heart rate (beats/min) (n = 433, 262, 171)	104 (24)	106 (25)	102 (22)	0.07
Temperature (°C) (n = 405, 244, 161)	37.6 (1.2)	37.6 (1.3)	37.7 (1.2)	0.84
P_a_O_2_/F_i_O_2_ (n = 496, 299, 197)	153 (96)	143 (94)	167 (98)	0.006
CK (U/L) (n = 230, 137, 93)	293 (91-835)	320 (102-949)	206 (90-681)	0.08
CK > 5000 IU/L (n = 230, 137, 93)	13 (5.7)	10 (7.3)	3 (3.2)	0.19
WBC (10^9^/L) (n = 409, 243, 166)	9.6 (6.5)	9.7 (6.8)	9.3 (6.0)	0.45
Albumin (g/L) (n = 275, 167, 108)	26.2 (7.0)	26.1 (7.1)	26.4 (7.0)	0.77
Platelets (10^9^/L) (n = 520, 313, 207)	188 (96)	184 (95)	194 (96)	0.26
Urea (mmol/L) (n = 76, 44, 32)	10.5 (21.0)	14.4 (26.9)	5.1 (2.8)	0.03
Creatinine (μmol/L) (n = 537, 326, 211)	124 (120)	155 (144)	76 (31)	<0.0001
Urine output (mL/24 hr) (n = 431, 270, 161)	1439 (1055)	1070 (939)	2058 (946)	<0.0001
**Clinical course and outcomes**				
Invasive mechanical ventilation	499 (88.8)	313 (91.5)	186 (84.6)	0.01
Inotropes or vasopressors	339 (60.3)	222 (64.9)	117 (53.2)	0.006
Antiviral treatment	530 (94.3)	326 (95.3)	204 (92.7)	0.20
Renal replacement therapy (n = 561, 342, 219)	85 (15.2)	85 (24.9)	0	<0.0001
Days of hospital stay (n = 523, 319, 204)	19.0 (12.0-35.0)	20.0 (12.0-40.0)	17.0 (12.0-30.0)	0.13
Days of ICU stay (n = 539, 325, 214)	11.0 (7.0-20.0)	12.0 (7.0-24.0)	10.0 (6.0-16.0)	0.002
Hospital mortality (n = 545, 329, 216)	113 (20.7)	85 (25.8)	28 (13.0)	0.0003

AKI is defined by the RIFLE Injury or Failure categories and no AKI by the RIFLE None or Risk categories.

Categorical data are presented as n (%) and continuous data as mean (SD) or median (Q1-Q3). For each variable, we specify (n) with data if it differs from the total in the first row.

### Incidence, characteristics, and predictors of AKI

Of 562 study patients, 342 (60.9%) developed AKI (worst RIFLE category Injury [n = 129, 23.0%] or Failure [n = 213, 37.9%]; Table 4). Using the 2 alternate approaches to estimate baseline kidney function, the incidence of AKI (Table 4) was lower (49.9%) if ICU day 1 creatinine was assumed as baseline and similar (61.2%) if the lowest ICU creatinine was assumed as baseline, with those receiving RRT designated as RIFLE Failure.

**Table 4:** Incidence of acute kidney injury in critically ill pH1N1 patients using different definitions of baseline renal function

**Primary analysis, with baseline glomerular filtration rate estimated as 75 ml/min/1**.**73 m**^**2**^	**Incidence**	**Hospital mortality**^**1, 2**^
	**(n** = **562)**	
No AKI	220 (39.1)	28/216 (13.0)
None	181 (32.2)	21/178 (11.8)
Risk	39 (6.9)	7/38 (18.4)
AKI	342 (60.9)	85/329 (25.8)
Injury	129 (23.0)	20/125 (16.0)
Failure	213 (37.9)	65/204 (31.9)
Renal replacement therapy, n = 561	85 (15.1)	26/84 (31.0)
Injury	3 (0.5%)	3/3 (100)
Failure	82 (14.6%)	26/81 (31.0%)
**Sensitivity analysis 1, assuming that first available serum creatinine is the baseline**	**n** = **537**	
No AKI	269 (50.1)	45/262 (17.2)
None	221 (41.2)	26/217 (12.0)
Risk	48 (8.9)	19/45 (42.2)
AKI	268 (49.9)	64/259 (24.7)
Injury	253 (47.1)	61/244 (25.0)
Failure	15 (2.8)	3/15 (20.0)
**Sensitivity analysis 2, assuming that lowest observed serum creatinine at any time is the baseline**	**n** = **562**	
No AKI	218 (38.8)	27/214 (12.6)
None	194 (34.5%)	25/191 (13.1)
Risk	24 (4.2%)	2/23 (8.7)
AKI	344 (61.2)	86/331 (26.0)
Injury	118 (21.0%)	15/114 (13.2)
Failure^3^	226 (40.2%)	71/217 (32.7)

Data are presented as n (%).

^1^ Denominators differ from the incidence column due to missing survival status (17/562 patients).

^2^ In the primary analysis, mortality differed significantly across the 4 RIFLE categories of None, Risk, Injury, and Failure (p < 0.0001) and across the categories of Risk, Injury, and Failure (p = 0.003).

^3^ For this analysis, patients who received RRT are assumed to have ‘Failure’.

In univariable analyses, AKI patients were more likely to be older (49.1 [15.1] vs. 46.4 [14.8] years, p = 0.03), obese (34.5% vs. 14.6%, p < 0.0001), diabetic (31.1% vs. 17.7%, p = 0.0003) and have CKD (12.9% vs. 1.8%, p < 0.0001) (Table 2). Similarly, AKI patients were also more severely ill on ICU day 1 (Table 3) compared to those without AKI, with higher APACHE II scores (23.0 [9.6] vs. 18.1 [7.6], p < 0.0001), SOFA scores (12.0 [3.8] vs. 9.7 [3.2], p < 0.0001), and worse PaO_2_/FiO_2_ ratios (143 [94] vs. 167 [98], p = 0.006). AKI patients had greater elevations in serum creatinine (155 [144] vs. 76 [31] μmol/L, p < 0.0001) and serum urea (14.4 [26.9] vs. 5.1 [2.8] mmol/L, p = 0.03) and lower 24-hour urine output (1070 [939] vs. 2058 [946] mL, p < 0.0001) on the first day of ICU admission.

Multivariable logistic regression (Table 5) showed that obesity (OR 2.94; 95% CI, 1.75-4.91), CKD (OR 4.50; 95% CI, 1.46-13.82), and increasing APACHE II score (OR per unit 1.06; 95% CI, 1.03-1.09) were independently associated with AKI. Higher P_a_O_2_/F_i_O_2_ ratio was protective (OR per 10-unit increase 0.98; 95% CI, 0.95-1.00).

**Table 5:** Logistic regression model for acute kidney injury in critically ill pH1N1 patients

**Variable**	**AKI**	**No AKI**	**Crude OR**	**P value**	**Adjusted OR**	**P value**
	**n** = **342**	**n** = **220**	**(95% CI)**		**(95% CI)**	
**H1N1 details, demographics, and comorbidities**						
Confirmed H1N1	291 (85.1)	188 (85.5)	0.97 (0.60-1.57)	0.90		
Age (yr)	49.1 (15.1)	46.4 (14.8)	1.01 (1.00-1.02)	0.03	1.00 (0.98-1.02)	0.98
Female sex	177 (51.8)	123 (55.9)	0.85 (0.60-1.19)	0.34	0.86 (0.56-1.32)	0.50
Ethnicity (n = 555, 336, 219)						
White/Caucasian (reference)	204 (60.7)	123 (56.2)	1.00		1.00	
Unknown/Mixed/ Other	68 (20.2)	49 (22.4)	0.84 (0.54-1.29)	0.42	0.80 (0.46- 1.39)	0.43
Aboriginal	47 (14.0)	27 (12.3)	1.05 (0.62-1.77)	0.86	1.01 (0.52-1.94)	0.98
Asian	17 (5.1)	20 (9.1)	0.51 (0.26-1.02)	0.06	0.82 (0.35-1.89)	0.64
Obesity	118 (34.5)	32 (14.6)	3.10 (2.00-4.79)	<0.0001	2.94 (1.75-4.91)	<0.0001
Diabetes mellitus	107 (31.3)	39 (17.7)	2.11 (1.40-3.20)	0.0004	1.61 (0.96-2.69)	0.07
Chronic obstructive pulmonary disease	56 (16.4)	42 (19.1)	0.83 (0.53-1.29)	0.41		
Alcohol abuse	41 (12.0)	33 (15.0)	0.77 (0.47-1.26)	0.30		
Chronic kidney disease	44 (12.9)	4 (1.8)	7.97 (2.82-22.5)	<0.0001	4.50 (1.46-13.82)	0.009
**Day 1 support, physiology, and laboratory values**						
APACHE II score (n = 508, 306, 202)	23.0 (9.6)	18.1 (7.6)	1.07 (1.04-1.09)	<0.0001	1.06 (1.03- 1.09)	<0.0001
Invasive mechanical ventilation (n = 553, 338, 215)	241 (71.3)	143 (66.5)	1.25 (0.86-1.81)	0.23		
Inotrope or vasopressor	164 (48.0)	87 (39.6)	1.41 (1.00-1.99)	0.05	1.29 (0.82-2.01)	0.27
Mean arterial pressure (mmHg) (n = 522, 315, 207)	73.4 (18.7)	75.9 (19.2)	0.93 (0.85-1.02)	0.14	0.95 (0.85-1.06)	0.32
P_a_O_2_/F_i_O_2_ (n = 496, 299, 197)	143 (94)	167 (98)	0.97 (0.96-0.99)	0.007	0.98 (0.95- 1.00)	0.04
Platelets (10^9^/L) (n = 520, 313, 207)	184 (95)	194 (96)	0.99 (0.97-1.01)	0.26		

Categorical data are presented as n (%) and continuous data as mean (SD). Heart rate, temperature, creatine kinase, WBC, albumin, and urea were excluded due to a high proportion of missing data. SOFA score was excluded because it was highly collinear with APACHE II score (Pearson r = 0.6). Urine output and creatinine were excluded because they were used to define the outcome. For each variable, we specify n when it differs from the totals in the first row. The final model included 442 patients and did not have evidence of lack of goodness of fit (p = 0.40). ORs for continuous variables refer to an increment of 1 unit (age, APACHE II) or 10 units (mean arterial pressure, P_a_O_2_/F_i_O_2_, platelets).

### Clinical course and outcomes

Patients with AKI were more likely to receive invasive mechanical ventilation and inotropes or vasopressors in the ICU, and had longer ICU but similar hospital length of stay (Table 3). Eighty-five patients received RRT (24.9% of patients with AKI and 15.1% of entire cohort); 82 achieved maximum RIFLE category of Failure and 3 achieved maximum RIFLE category of Injury prior to RRT initiation. Multivariable logistic regression showed that independent predictors of receiving RRT (Table 6) were obesity (OR 2.25; 95% CI, 1.14-4.44), day 1 mechanical ventilation (OR 4.09; 95% CI, 1.21-13.84), and increasing APACHE II score (OR per unit, 1.07; 95% CI, 1.03-1.12) and day 1 creatinine (OR per 10 μmol/L, 1.06; 95% CI, 1.03-1.10).

**Table 6:** Logistic regression model for renal replacement therapy in critically ill H1N1 patients

**Variable**	**RRT**	**No RRT**	**Crude OR**	**P value**	**Adjusted OR**	**P value**
	**n** = **85**	**n** = **476**	**(95% CI)**		**(95% CI)**	
**Demographics and comorbidities**						
Age (yr)	46.2 (13.1)	48.4 (15.3)	0.99 (0.98-1.01)	0.21	0.98 (0.96-1.00)	0.07
Female sex	43 (50.6)	256 (53.8)	0.88 (0.55-1.40)	0.59	1.81 (0.91-3.58)	0.09
Ethnicity (n = 554, 85, 469)						
White/Caucasian (reference)	56 (65.9)	271 (57.8)	1.00		1.00	
Unknown/Other	19 (22.4)	97 (20.7)	0.95 (0.54-1.68)	0.85	0.53 (0.22-1.28)	0.16
Aboriginal	6 (7.1)	68 (14.5)	0.43 (0.18-1.03)	0.06	0.40 (0.13-1.20)	0.10
Asian	4 (4.7)	33 (7.0)	0.59 (0.20-1.7)	0.33	1.11 (0.31-3.95)	0.88
Obesity	33 (38.8)	117 (24.6)	1.95 (1.20-3.16)	0.007	2.25 (1.14-4.44)	0.02
Diabetes mellitus	23 (27.1)	123 (25.8)	1.06 (0.63-1.79)	0.81		
Chronic obstructive pulmonary disease	5 (5.9)	93 (19.5)	0.26 (0.10-0.65)	0.004	0.26 (0.06-1.20)	0.08
Alcohol abuse	10 (11.8)	64 (13.5)	0.86 (0.42-1.75)	0.67		
Chronic kidney disease	15 (17.7)	33 (6.9)	2.88 (1.49-5.57)	0.002	0.85 (0.28-2.57)	0.78
**Day 1 support, physiology, and laboratory values**						
APACHE II score (n = 507, 80, 427)	27.6 (9.2)	19.8 (8.6)	1.10 (1.07-1.13)	<0.0001	1.07 (1.03-1.12)	0.0009
Invasive mechanical ventilation (n = 552, 85, 467)	72 (84.7)	311 (66.6)	2.78 (1.49-5.17)	0.001	4.09 (1.21-13.84)	0.02
Inotrope or vasopressor	44 (51.8)	206 (43.3)	1.41 (0.89-2.23)	0.15	0.78 (0.37-1.61)	0.50
Mean arterial pressure (mmHg) (n = 521, 79, 442)	70.4 (16.9)	75.2 (19.2)	0.87 (0.76-0.995)	0.04	0.97 (0.81–1.16)	0.75
P_a_O_2_/F_i_O_2_ (n = 495, 76, 419)	132 (95)	156 (96)	0.97 (0.94-1.00)	0.046	0.97 (0.93-1.01)	0.16
Platelets (10^9^/L) (n = 519, 76, 443)	164 (84)	192 (96)	0.97 (0.94-0.995)	0.02	0.99 (0.95-1.03)	0.64
Creatinine (μmol/L) (n = 536, 79, 457)	233 (185)	105 (93)	1.07 (1.05-1.09)	<0.0001	1.06 (1.03-1.10)	<0.0001

Categorical data are presented as n (%) and continuous data as mean (SD). Heart rate, temperature, creatine kinase, WBC, albumin, urea, and urine output were excluded due to a high proportion of missing data. SOFA score was excluded because it was highly collinear with APACHE II (Pearson r = 0.6). For each variable, we specify n when it differs from the totals in the first row. The final model included 411 patients and did not have evidence of lack of goodness of fit (p = 0.17). ORs for continuous variables refer to an increment of 1 unit (age, APACHE II), 10 units (mean arterial pressure, P_a_O_2_/F_i_O_2_, platelets, creatinine), or 100 units (urine output).

Hospital survival status was available for 545 patients (97.0%). When stratified by AKI severity (Table 4), crude mortality differed (p < 0.0001) across RIFLE categories of None (21/178, 11.8%), Risk (7/38, 18.4%), Injury (20/125, 16.0%), and Failure (65/204, 31.9%). Mortality in the AKI and no AKI categories was similar among methods used to estimate baseline renal function (Table 4). Univariable analysis showed that AKI was associated with hospital mortality, but this association was no longer significant after multivariable adjustment (OR 1.35; 95% CI, 0.74-2.48; Table 7). Independent predictors of increased hospital mortality were age (OR 1.02 per year; 95% CI, 1.00-1.04) and APACHE II score (OR 1.05 per unit; 95% CI, 1.01-1.08), whereas higher P_a_O_2_/F_i_O_2_ ratio (OR 0.95 per 10 units; 95% CI, 0.92-0.99) and platelets (OR 0.92 per 10 × 10^9^/L; 95% CI, 0.89-0.96) on day 1 were associated with lower mortality.

**Table 7:** Logistic regression model for hospital mortality in critically ill H1N1 patients

**Variable**	**Deaths**	**Survivors**	**Crude OR**	**P value**	**Adjusted OR**	**P value**
	**(n = 113**)	**(n = 432)**	**(95% CI)**		**(95% CI)**	
**H1N1 details**, **demographics**, **and comorbidities**						
Confirmed H1N1	104 (92.0)	364 (84.3)	2.16 (1.04-4.47)	0.04	1.59 (0.66-3.80)	0.30
Days of symptoms before hospitalization	5.5 (5.5)	5.2 (4.6)	1.01 (0.97-1.06)	0.55		
Days of symptoms before ICU admission (n = 534, 110, 424)	7.2 (6.5)	6.2 (5.1)	1.03 (1.00-1.07)	0.08	1.04 (1.00-1.09)	0.06
Age (yr)	51.8 (15.6)	47.0 (14.6)	1.02 (1.01-1.04)	0.003	1.02 (1.00-1.04)	0.03
Female sex	55 (48.7)	234 (54.2)	0.80 (0.53-1.22)	0.30	1.00 (0.58-1.71)	0.99
Obesity	42 (37.2)	106 (24.5)	1.82 (1.17-2.82)	0.008	1.49 (0.85-2.62)	0.16
Diabetes mellitus	24 (21.2)	116 (26.9)	0.74 (0.45-1.21)	0.23		
Chronic obstructive pulmonary disease	20 (17.7)	75 (17.4)	1.02 (0.60-1.76)	0.93		
Alcohol abuse	9 (8.0)	64 (14.8)	0.50 (0.24-1.03)	0.06	0.77 (0.34-1.75)	0.54
Chronic kidney disease	15 (13.3)	33 (7.6)	1.85 (0.97-3.54)	0.06	1.35 (0.50-3.61)	0.55
**Day 1 support**, **physiology**, **and laboratory parameters**						
APACHE II (n = 497, 101, 396)	25.0 (10.2)	20.0 (8.6)	1.06 (1.04-1.09)	<0.0001	1.05 (1.01-1.08)	0.006
Invasive mechanical ventilation (n = 537, 111, 426)	82 (73.9)	289 (67.8)	1.34 (0.84-2.14)	0.22		
Inotrope or vasopressor	52 (46.0)	186 (43.1)	1.13 (0.74-1.71)	0.57		
Mean arterial pressure (mmHg) (n = 506, 103, 403)	75.1 (20.9)	74.2 (18.6)	1.02 (0.91-1.14)	0.70		
P_a_O_2_/F_i_O_2_ (n = 480, 101, 379)	126 (84)	161 (99)	0.95 (0.93-0.98)	0.002	0.95 (0.92-0.99)	0.007
Creatinine (μmol/L) (n = 521, 109, 412)	159 (127)	115 (118)	1.03 (1.01-1.04)	0.002	1.001 (0.98-1.03)	0.95
Platelets (10^9^/L) (n = 504, 105, 399)	151 (93)	196 (91)	0.94 (0.91-0.97)	<0.0001	0.92 (0.89-0.96)	<0.0001
**Other**						
Acute kidney injury	85 (75.2)	244 (56.5)	2.34 (1.47-3.73)	0.0004	1.35 (0.74-2.48)	0.33

Categorical data are presented as n (%) and continuous data as mean (SD). Mortality status was missing for 17 patients. Heart rate, temperature, creatine kinase, WBC, albumin, urea, and urine output were excluded due to a high proportion of missing data. SOFA score was excluded because it was highly collinear with APACHE II (Pearson r = 0.6). For each variable, we specify n when it differs from the first row total. The final model included 414 patients and did not have evidence of lack of goodness of fit (p = 0.49). ORs for continuous variables refer to an increment of 1 unit (age, APACHE II) or 10 units (mean arterial pressure, P_a_O_2_/F_i_O_2_, platelets, creatinine).

## Discussion

### Summary of major findings

We performed a prospective multi-centre cohort study in representative ICUs from across Canada of patients with confirmed or probable pH1N1 infection, the majority of whom were mechanically ventilated, to describe the incidence of AKI, rate of RRT utilization, and associated outcomes. AKI was common, complicating the course of 60.9% of patients. Most developed more severe forms of AKI, defined by RIFLE categories of Injury and Failure. Moreover, 24.9% of those with AKI, or 15.1% of the entire pH1N1 cohort, received RRT. In general, we found that AKI was more likely to occur in older patients with a higher burden of comorbid disease, specifically obesity, diabetes mellitus, and CKD. In addition, those developing AKI presented with higher acuity of illness, greater burden of organ dysfunction and showed worse kidney function early after ICU admission compared to those not developing AKI. Finally, pH1N1 patients whose course was complicated by AKI received far greater intensity (i.e. invasive mechanical ventilation, vasoactive support, and RRT) and duration of support in ICU, with comparable length of hospital stay and mortality.

### Strengths and limitations

Our study has several strengths. It provides prospective, multicentre, nationally representative data of critically ill patients with pH1N1 and describes the incremental burden associated with development of AKI. Our study also provides complementary data to other large observational studies from distinct geographic regions on the attributable burden of AKI in pH1N1-related critical illness [1,5,23,25]. However, we also recognize our study’s limitations. First, despite a large cohort, data on kidney function were missing or unavailable for 16.2% of patients. These excluded patients were less severely ill and less intensively treated, and thus we may have overestimated the incidence of AKI. Second, we assumed normal baseline renal function among included patients and may thus have overestimated the incidence of AKI, but the incidence of AKI and mortality among these patients did not dramatically change in sensitivity analyses that would be expected to underestimate the incidence. Only 24-hr daily urine output, as opposed to hourly urine output, was available for determination of RIFLE category. Therefore, we modified our criteria as previously reported [34], possibly underestimating the incidence of AKI by missing patients with transient decrements in urine output. Third, we may not have accounted for additional confounding variables in the associations of clinical characteristics and AKI, RRT, and hospital mortality. In particular, we did not have information on the type or amount of fluid resuscitation, non–H1N1-related infections, or other nephrotoxins. We also did not collect data on the modality, mode, or dose of RRT, although none of these has been shown to consistently influence mortality [35]–[37].

### Comparison with prior literature

In our study, the incidence of AKI complicating the course of pH1N1-related critical illness in Canada was higher than reported from Spain (17.7%) [23], South Korea (22.6%) [19], Australia and New Zealand (34.0%) [26], United States (42.0%) [16], and similar to that reported in Argentina (51.0%) [24,25]. Many risk factors for AKI identified in our cohort, specifically older age [15,19], diabetes [19,23,26], CKD [16,19,26], and illness severity [13,19,23,25,26] have been consistently shown to predict AKI in prior studies. Other studies have also identified male sex [23], pregnancy [26], immunosuppression [19], hypertension [19], and co-infection [23] as risk factors.

Not only was AKI common in our cohort, but most patients with AKI progressed to the RIFLE category of Failure (62.3%), similar to previous investigations [19,23,25]. Rates of RRT utilization among all pH1N1 patients (15.1%) and among those developing AKI (24.9%) were also relatively high and within the ranges (7.6-23.8% and 22.0-44.0%, respectively) described previously [19,23,25,27]. These data contrast with large observational studies in non-pandemic populations that have generally shown that only 4-6% of critically ill patients receive RRT [38]. The higher rate of RRT utilization in pH1N1-related critical illness may be explained by near universal lung injury and higher acuity of illness in this population, which may increase clinicians’ desire to mitigate fluid accumulation and attempt extracorporeal removal of extravascular lung water. Other reasons for variation in RRT utilization among pH1N1 patients may include differences among regions and clinicians in the perceived indications, utilization, and timing of initiation of RRT.

Obesity has been found to be highly prevalent among critically ill patients with pH1N1-related respiratory failure [2,5,39]. Whether obesity identifies poorer general health characterized by cardiovascular disease, diabetes, or maladaptive immune function, or contributes independent risk due to alterations in pulmonary function and diminished reserve, remains uncertain. Obesity was associated with 3-fold higher odds of AKI, confirmed in other studies [16,23,25].

Elevated creatine kinase (CK) has been implicated as a contributing factor for AKI in pH1N1 [14,17,22,28]. Pettila et al. reported that 15.8% of AKI patients had CK levels exceeding 5000 IU/L and found a trend of increasing CK levels with worsening AKI [26]. Demirjian et al. found higher median CK levels in AKI compared with non-AKI patients, with peak CK >2000 IU/L occurring 22% of those with AKI and in no patient without AKI [16]. In our study, CK levels were higher in AKI compared with non-AKI patients; however, this difference was not statistically significant. While rhabdomyolysis may have a supporting causal role in AKI among patients with pH1N1-related critical illness, the etiology is likely to be multi-factorial. Recent data have suggested that acute glomerular disease or active viral replication in renal epithelial tissue may also contribute [20,24].

From a health policy and planning perspective, anticipating the need for additional resources for a pandemic such as pH1N1 [6], with a relatively high attack rate and propensity for organ failure, is vital. In the case of pH1N1, needs included additional ICU bed capacity, availability of ventilators, and access to ECLS [9,10]. In addition, pH1N1 patients had greater relative utilization of RRT, highlighting the importance of expanded capacity for RRT services. Each of these technologies would be expected to increase costs related to staffing, disposables, and length of stay. This added resource burden may contribute to decreased ICU bed availability and hospital surge capacity during periods of heightened demand and complicate triage decision-making during future pandemics [40].

## Conclusions

In summary, we describe the incidence and outcomes associated with AKI complicating the course of pH1N1-related critical illness in a large Canadian cohort. AKI occurred in approximately 60% of patients, with the majority developing more advanced AKI, of whom a large minority (24.9%) received RRT. Development of AKI had important implications for utilization of health resources, generally portending a significantly longer duration of ICU stay; however, it was not an independent predictor of hospital mortality.

## Abbreviations

APACHE: Acute physiology and chronic health evaluation; AKI: Acute kidney injury; CI: Confidence interval; CK: Creatine kinase; CKD: Chronic kidney disease; ECLS: Extracorporeal life support; ICU: Intensive care unit; PaO2/FiO2: Partial pressure of arterial oxygen/fractional concentration of inspired oxygen; OR: Odds ratio; pH1N1: Pandemic influenza A (H1N1); RIFLE: Risk, injury, failure, loss, end-stage renal disease classification; RRT: Renal replacement therapy; SOFA: Sequential organ failure assessment; WBC: White blood cell count.

## Competing interests

The authors declared that they have no competing interests.

## Authors’ contributions

RAF led the Canadian H1N1 observational study. SMB, MMS, and RAF conceived of this study. SMB, RAF, and NKJA designed the analyses. JL analyzed the data. SMB wrote the first draft of the manuscript and revised it. NKJA supervised the data analyses and revised the manuscript. MMS and RAF further revised the manuscript. All authors approved the final version.

## Pre-publication history

The pre-publication history for this paper can be accessed here:

http://www.biomedcentral.com/1471-2369/14/123/prepub

## Supplementary Material

Additional file 1 List of members of the Canadian Critical Care Trials Group H1N1 Collaborative. Click here for file
