# Supplementary material for: Acute kidney injury among critically ill patients with pandemic H1N1 influenza A in Canada: cohort study
Source: BMC Nephrol. 2013 Jun 13;14:123. doi: 10.1186/1471-2369-14-123 (PMC3694036; doi:10.1186/1471-2369-14-123)
Supplement: Additional file 1 — List of members of the Canadian Critical Care Trials Group H1N1 Collaborative. [file 1471-2369-14-123-S1.pdf]

Members of the Canadian Critical Care Trials Group H1N1 Collaborative

Bandrauk N, Leonard S, Moores E, Barter J, Fiander J, Hall R, Green R, Heinzler D, Julien L, Wright D, Soder C, Earle R, King S, Lauzier F, Turgeon A, Roy C, Gagne C, Lellouche F, Ferland M-C, Dussault P, Poirier G, Lamontagne F, Neas I, Masse V, Yale P, Albert M, Arsenault I, Deroy P, Laporta D, Hornstein D, Verhoef B, Khwaja K, Banici L, Ahern SP, Skrobic Y, Harvey J, Santschi M, Ducharme-Crevier L, Jouvet P, Lacroix J, Withington D, Al Otaibi T, Dugas M-A, St-Pierre L, Cassista J, Gosselin L, McIntyre L, Gaudet C, Saginur R, Pagliarello J, Watpool I, Mcardle T, Foxall J, Murphy E, Bergeron C, Lewis MJ, Muscedere J, Godfrey N, Fleury M, Hollinger G, Malus E, Williams N, Hrytsyk M, Meade M, Spoto C, Freitag A, Choong K, Irwin N, Karachi T, Lapinsky S, Ferguson N, Ethier C, Stewart T, Lubchansky S, Cook DJ, MacDonald E, Wilton K, Tkaczyk A, Kho M, Sivaloganathan L, Cirone R, Marshall J, Burns K, Smith O, Friedrich J, Fry B, Porretta K, Fowler R, Long J, Pinto R, Adhikari NKJ, Marinoff N, Maher A, Moran C, Delaney J,. Singh JM, Herridge M, Granton J, Brockest N, Matte A, Tansey C, Chu L, Dennis M, di Nino M, McRitchie D, Correa J-A, Martin C, Campbell E, Menon K, Creery D, Doherty D, Dhanani S, Ward R, Alsaati B, Choong K, Duffett M, Irwin N, Caceres TL, Hutchison J, Keating L, Gaiteiro R, Krancevic A-M, Van Huyse J, Kornecki A, Foster J, Kukreti B, Boyle D, Kostiw K, Green L, Fung D, Jacko N, Herzog J, Berg G, Bettello P, Ross A, Marcello N, Agarwala R, Anderson W, Stoger S, Kumar A, Zarychanski R, Siddiqui F, Sharma S, Wiebe K, Ramsey C, Olafson K, Funk D, Garland A, Janz W, Marten N, Siddiqui M, Veroukis S, Kesselman M, Angelo L, Wali Ahsan M, Penner C, Wong A, Shaw S, Friedt J, Wolffe G, Thomson S, Holt T, Andreychuk B, Stelfox T, Bobranska-Artiuch B, Heule M, Kutsogiannis D, Johnston C, Bartel R, Thompson P, Jossy D, Norris S, Stollery DE, MacLure T, Hardy-Joel R, Barchard J, Bagshaw SM, Irwin M, Joffe A, Sheppard C, Gresiuk C, Hodson L, Gilfoyle E, Menzies K, Dodek P, Ashley BJ, Dunham H, Griesdale D, Foster D, Gardner M, Logie S, Reynolds S, Keenan S, Mans S, Palmer J, Svetik M, Wood G, Atkins L, Aldred- Portman B, Skippen P, Kissoon N, Krahn G, Burzynski J, Barclay A, Patel A, Kandola K, Pender K, Hanley B, Braden KD, Beckett R, Bilton P.
